# Hepatic B‐Cell Lymphoma in a Common Mynah (*Acridotheres tristis*): A Case Report

**DOI:** 10.1155/crve/5662048

**Published:** 2026-10-07

**Authors:** Pouneh Hajipour, Yousef Toutounkar, Navid Khosroabadi, Omid Dezfoulian, Jamshid Razmyar

**Affiliations:** ^1^ Department of Avian Diseases Faculty of Veterinary Medicine University of Tehran Tehran Iran ut.ac.ir; ^2^ Department of Bacterial Diseases Sana Institute for Avian Health and Diseases Research Tehran Iran; ^3^ Department of Veterinary Radiology Sana Research Institute of Avian Diseases and Health Tehran Iran; ^4^ Department of Pathobiology School of Veterinary Medicine Lorestan University Khorram Abad Iran lu.ac.ir

**Keywords:** avian oncology, companion bird, hepatomegaly, immunohistochemistry, round-cell neoplasm

## Abstract

**Background:**

Hepatic lymphoma is rarely characterized in mynah birds, and severe hepatomegaly in this group is often interpreted in relation to iron storage disease or other metabolic hepatopathies.

**Case Description:**

This report describes hepatic B‐cell lymphoma in a 14‐year‐old common mynah (*Acridotheres tristis*) presented with lethargy, altered vocalization, poor body condition, ascites, dyspnea, chronic ataxia, and left‐leg lameness. Radiography revealed a large soft‐tissue opacity in the middle coelomic cavity, with caudodorsal displacement of the intestinal loops and compression of the thoracoabdominal air sacs. Ultrasonography identified a very large hepatic mass with granular and mixed echogenicity occupying most of the coelomic cavity. The bird was euthanized at the owner′s request because of the severity of the lesion and poor clinical condition. At necropsy, the liver was markedly enlarged, dark red to reddish‐brown, mottled and occupied most of the coelomic cavity. Histologically, the hepatic parenchyma was extensively replaced by densely packed mononuclear neoplastic cells, with effacement of hepatic cords and hepatocellular necrosis. No neoplastic infiltration was identified in the examined tissues, including spleen, kidney, intestine, lung, and heart; therefore, the liver was the only confirmed site of lymphoma among the examined tissues in this case. An immunohistochemical and histochemical panel including CD3, CD79*α*/MB‐1, CD20, lysozyme, CD68, alpha‐1‐antitrypsin, toluidine blue, and Giemsa staining was applied to characterize the tumor lineage and exclude other round‐cell neoplasms. Neoplastic cells showed diffuse cytoplasmic immunoreactivity for CD79*α*/MB‐1 and lacked evidence of T‐cell, monocyte/macrophage, or mast cell differentiation, supporting a diagnosis of hepatic B‐cell lymphoma.

**Conclusion:**

This case highlights the need for tissue‐based diagnosis in mynah birds with marked hepatomegaly and demonstrates the value of integrating diagnostic imaging, histopathology, immunohistochemistry, and molecular testing in avian hepatic round‐cell tumors.

## 1. Introduction

Neoplastic diseases are increasingly recognized in domestic and companion birds, but their true prevalence, biological behavior, and species distribution remain incompletely characterized, particularly in less commonly studied avian groups [1]. Mynah birds, including the common mynah (*Acridotheres tristis*), are widely kept as companion and ornamental birds in many regions; however, published information on their neoplastic diseases remains limited compared with psittacine and galliform birds. In mynahs, hepatic lesions are of particular diagnostic interest because liver enlargement or diffuse parenchymal alteration may arise from diverse pathological processes, including metabolic, inflammatory, and neoplastic conditions [2].

Lymphoma is a malignant round cell neoplasm of lymphoid origin that may occur in birds as either localized or multicentric disease. Visceral involvement can include the liver, spleen, kidneys, gastrointestinal tract, and other organs [3, 4]. Most published data on avian lymphoma are derived from psittacine birds, in which the liver is among the most frequently affected organs and B‐cell lymphoma represents the predominant immunophenotype. Reports in other companion birds further indicate that hepatic involvement may be a major component of both B‐cell and T‐cell lymphomas. The pathogenesis of lymphoma in birds is considered multifactorial and may involve extrinsic factors, including oncogenic viruses such as avian leukosis virus (ALV), reticuloendotheliosis virus (REV), and herpesviruses, as well as environmental exposures and intrinsic host factors such as age and genetic susceptibility. Clinically, avian lymphoma is often nonspecific and may present with lethargy, weight loss, anorexia, vomiting, dyspnea, abdominal distension, hepatomegaly, or neurological signs, making early diagnosis challenging [4, 5].

Despite increasing recognition of avian lymphoid neoplasms, hepatic lymphoma in mynah birds remains poorly characterized. This diagnostic gap is important because hepatic lymphoid infiltrates may be difficult to distinguish from inflammatory hepatopathy, iron‐associated metabolic liver disease, or primary epithelial hepatic tumors such as cholangiocellular carcinoma on routine histology alone [2, 6]. Therefore, accurate diagnosis requires correlation of clinical findings, imaging, histopathology, and immunohistochemistry. To the authors′ knowledge, this is the first immunohistochemically supported report of hepatic B‐cell lymphoma in a common mynah (*A. tristis*).

## 2. Case Presentation

A 14‐year‐old male common mynah (*A. tristis*), weighing 105 g, was presented for evaluation of progressive coelomic enlargement and suspected hepatic disease. The bird had a short history of lethargy and altered vocalization. Ataxia and left‐leg lameness had also been reported before presentation. Appetite was preserved according to the available clinical information. On physical examination, the bird was in poor body condition and showed marked ventral coelomic distension. Abnormal respiratory movements were observed, consistent with a space‐occupying coelomic lesion and reduced air‐sac capacity. The bird was maintained on a mynah‐appropriate low‐iron diet (Oshkaya Mynah Food, Oshkaya Pet Food Co., Iran) with selected fruit and vegetable supplementation, and had no history of previous medication before presentation. Supportive management was initiated, including coelomic fluid drainage and fluid therapy.

Whole‐body lateral radiography revealed a large soft‐tissue opacity in the middle coelomic cavity. The lesion displaced the intestinal loops caudodorsally and compressed the thoracoabdominal air sacs. Coelomic ultrasonography identified a very large hepatic mass (53 × 45 mm) with granular and mixed echogenicity occupying a major portion of the coelomic cavity. The imaging findings suggested a hepatic neoplastic process. Iron storage disease and other nonneoplastic hepatopathies were also considered as differential diagnoses. However, the mass‐like appearance of the lesion, severe coelomic displacement, and compression of adjacent viscera favored a neoplastic process. Definitive characterization required histopathological and immunohistochemical examination (Figure 1).

**Figure 1:**
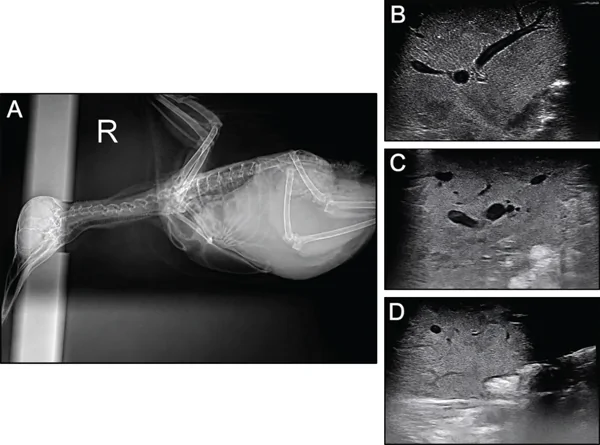
Diagnostic imaging of hepatic B‐cell lymphoma in a common mynah (*Acridotheres tristis*). (A) Lateral radiograph showing a large midcoelomic soft‐tissue opacity with intestinal displacement and thoracoabdominal air‐sac compression. (B–D): B‐mode coelomic ultrasonography showing marked hepatomegaly with a large heterogeneous to mixed‐echogenic mass‐like hepatic lesion occupying a major portion of the coelomic cavity.

Additional antemortem diagnostic procedures, including fine‐needle aspiration, hematological evaluation, and serum biochemical analysis, were not performed because of the bird′s critical clinical condition and the owner′s decision not to pursue additional diagnostic procedures. Because of the severity of the hepatic lesion and the poor clinical condition, the bird was euthanized at the owner′s request and submitted for necropsy. Before opening the coelomic cavity, the ventral body wall was markedly distended and tense, producing a rounded abdominal contour (Figure 2A). After opening the coelomic cavity, the liver was severely enlarged and occupied most of the coelomic space, causing marked displacement and compression of adjacent viscera (Figure 2B). Macroscopically, the hepatic lesion appeared as a large, expansile, dark red to reddish‐brown, fleshy‐appearing mass replacing most of the liver. The enlarged liver measured approximately 9–10 cm in length and 3–4 cm in maximal width/thickness. The capsular surface was smooth to mildly irregular, glistening, and diffusely mottled, with multifocal pale red, dark red and brown discoloration. The lesion was not grossly encapsulated. Multifocal darker areas were present on the hepatic surface, compatible with congestion, hemorrhage or necrosis on gross inspection. After removal from the coelomic cavity, the liver remained markedly enlarged and elongated, with an irregular mottled capsular surface (Figure 2C).

**Figure 2:**
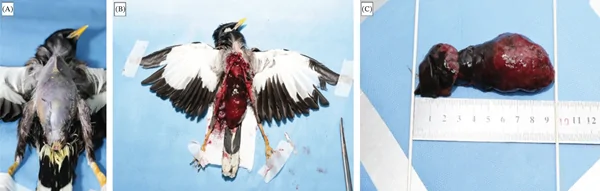
Gross findings of hepatic B‐cell lymphoma in a common mynah (*Acridotheres tristis*). (A) Marked ventral coelomic distension before necropsy. (B) Severe hepatomegaly occupying most of the coelomic cavity. (C) Excised mottled liver, approximately 10 cm in length, with a glistening dark red to reddish‐brown capsular surface.

Representative samples from the liver and other tissues, including spleen, kidney, intestine, lung, and heart were collected for histopathological evaluation. Additional hepatic tissue was submitted for immunohistochemistry and molecular testing. Tissue samples were fixed in 10% neutral buffered formalin, processed routinely, embedded in paraffin, sectioned and stained with hematoxylin and eosin [7]. Microscopic examination of the liver showed extensive infiltration of the hepatic parenchyma by mononuclear neoplastic cells. The infiltrate formed focal to coalescing and diffuse aggregates within the liver. The neoplastic population caused progressive hepatocellular destruction and was associated with hepatocellular necrosis (Figure 3A). In severely affected areas, hepatic cords were partially to completely effaced, and only small residual clusters of hepatocytes remained between neoplastic cells. The tumor cells were small, basophilic, and densely arranged, with compact hyperchromatic nuclei and scant cytoplasm (Figure 3B). No extrahepatic neoplastic infiltration was detected in the tissues examined; therefore, the liver was the only confirmed site of lymphoma in this case. These findings supported a destructive hepatic round cell neoplasm rather than a primary epithelial hepatic tumor [3].

**Figure 3:**
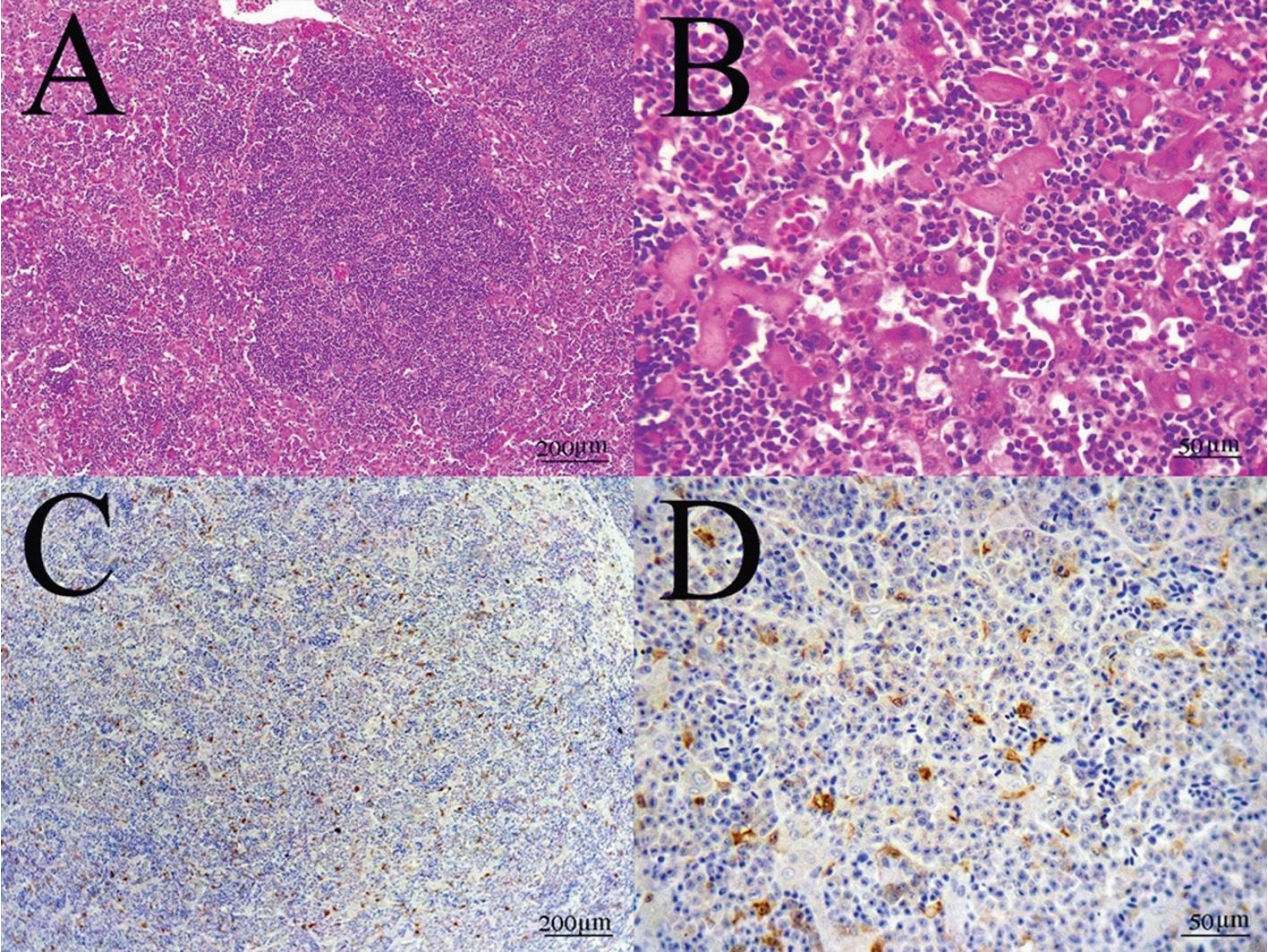
Histopathological and immunohistochemical findings of hepatic lymphoma in a common mynah (*Acridotheres tristis*). **(**A) Liver, hematoxylin and eosin staining. The hepatic parenchyma is extensively infiltrated by numerous mononuclear neoplastic cells, resulting in progressive hepatocellular destruction and necrosis. The neoplastic cells are small, basophilic and arranged in focal to coalescing and diffuse aggregates. (B) Liver, hematoxylin and eosin staining. Hepatic cords are partially to completely effaced by neoplastic cells, with only small residual clusters of hepatocytes remaining among densely packed mononuclear cells. The tumor cells have compact, strongly basophilic nuclei and scant cytoplasm. (C) Liver, CD79*α* immunohistochemistry. Neoplastic cells show widespread immunolabeling throughout the lesion. (D) Liver, CD79*α* immunohistochemistry. Strong cytoplasmic immunolabeling is evident in the tumor cell population.

Immunohistochemistry staining on liver was performed using the following antibodies: polyclonal rabbit anti‐CD3 (EP41, Vitro master Diagnostica, Spain), 1:100 CD79*α*/MB‐1 (SP18, Vitro master), 1:100 Lysozyme (orb1729844, Biorbyt, United Kingdom), 1:50, Alpha1‐antitrypsin (orb1712691, Biorbyt) 1:100, and mouse monoclonal antibody against CD20 (Vitro master, L26) 1:50, CD68 (Vitro master, KP‐1) 1:100 (Table 1).

**Table 1:** Immunohistochemical and histochemical panel used for characterization of the hepatic round‐cell neoplasm in a common mynah (*Acridotheres tristis*).

Marker/stain	Clone/catalog and manufacturer	Dilution	Antigen retrieval	Tumor‐cell result	Internal/positive control	Interpretation
CD3	EP41, Vitro Master Diagnostica, Spain	1:100	Tris–EDTA, pH 9	Negative in neoplastic cells; scattered background T lymphocytes only	Thymic lymphocytes positive	Does not support T‐cell phenotype
CD79*α*/MB‐1	SP18, Vitro Master Diagnostica, Spain	1:100	Tris–EDTA, pH 9	Diffuse cytoplasmic immunolabeling in neoplastic lymphoid cells, moderate to strong intensity	Bursal B cells positive	Supports B‐cell lineage
CD20	L26, Vitro Master Diagnostica, Spain	1:50	Tris–EDTA, pH 9	No diagnostic immunolabeling in neoplastic cells	Bursal B cells positive	CD20 negativity interpreted cautiously and did not exclude B‐cell lineage because CD79*α*/MB‐1 was diffusely positive
CD68	KP‐1, Vitro Master Diagnostica, Spain	1:100	Tris–EDTA, pH 9	Negative in neoplastic cells; scattered background phagocytic cells only	Macrophage/phagocytic cells positive	Does not support histiocytic/macrophage origin
Lysozyme	orb1729844, Biorbyt, United Kingdom	1:50	Tris–EDTA, pH 9	Negative in neoplastic cells; scattered background phagocytic cells only	Background/control phagocytic cells positive	Does not support myeloid/macrophage differentiation
Alpha‐1‐antitrypsin	orb1712691, Biorbyt	1:100	Tris–EDTA, pH 9	Negative in neoplastic cells	Background/control myeloid‐lineage cells	Does not support histiocytic/myeloid origin
Toluidine blue	—	—	Not applicable	Negative; no metachromatic cytoplasmic granules in tumor cells	Mast cells/internal positive control	Does not support mast‐cell tumor
Giemsa	—	—	Not applicable	Negative for mast‐cell‐type cytoplasmic granules in tumor cells	Mast cells/internal positive control	Does not support mast‐cell tumor

Histochemical staining for toluidine blue (TB) and Giemsa stain was also performed. The sections were deparaffinized, rehydrated, and treated in a target retrieval solution of Tris–EDTA (pH = 9). For antigen retrieval, samples containing the target retrieval solution were put in a 98°C heating bath and kept there for 20 min. To block endogenous peroxidase activity, samples were treated with hydrogen peroxide (3% H2O2) in PBS for 15 min, and normal rabbit serum (5%) in PBS was used to avoid nonspecific background staining. The samples were incubated with primary antibodies for 1 h. Secondary antibody staining using a goat antirabbit biotinylated antibody and goat antimouse (prediluted, Biocare, United States) for polyclonal and monoclonal, respectively, for 20 min was used to identify primary antibodies. Then, 20‐min incubation with prediluted streptavidin horseradish peroxidase (sHRP) (Biocare, United States) was done. Finally, DAB was used as a chromogen to observe antibody binding areas, and Mayer′s Hematoxylin (Bio Optica, Italy) was used to counterstain the samples. Avian lymphoid tissues, including spleen, thymus and bursa of Fabricius, were used as positive controls. The primary antibody was replaced by nonimmune serum used as negative control.

Positive‐control tissues showed the expected immunolabeling patterns, supporting antibody reactivity in avian lymphoid tissues. The immunohistochemical and histochemical findings are summarized in Table 1. Briefly, the hepatic neoplastic lymphoid cells showed diffuse cytoplasmic CD79*α*/MB‐1 immunolabeling with moderate intensity, whereas CD3, CD20, CD68, lysozyme, and alpha‐1‐antitrypsin did not show diagnostic staining in the neoplastic population. TB and Giemsa staining did not demonstrate metachromatic cytoplasmic granules. Overall, this profile supported B‐cell lymphoid differentiation and did not support T‐cell, macrophage/histiocytic, myeloid, or mast cell origin (Figure 3C,D).

Polymerase chain reaction (PCR) testing was performed to investigate the possible presence of selected avian oncogenic viruses potentially associated with lymphoid neoplasia, including Marek′s disease virus type 1 (MDV‐1), ALV, and REV. A liver sample was processed for PCR. Briefly, the sample was homogenized in sterile PBS, subjected to three freeze–thaw cycles, and centrifuged at 12,000 × *g* for 20 min [8, 9]. The clarified supernatant was used for viral nucleic acid extraction using the BioGene Viral DNA/RNA Extraction Kit according to the manufacturer′s instructions. Extracted nucleic acids were screened for MDV‐1/GaHV‐2, ALV, and REV. MDV‐1 was tested by PCR targeting the pp38 gene using primers described by Cao et al. [10]. ALV and REV were tested by multiplex PCR targeting the p27 gene and LTR region, respectively, using primers according to Gopal et al. [11]. Appropriate positive and negative controls were included in each PCR run. Archived positive control samples previously confirmed by PCR for the corresponding target viruses were used as positive controls, and nuclease‐free water was used as the negative control. No viral nucleic acid was detected for the tested agents.

Based on the clinical presentation, imaging evidence of a large hepatic mass, gross severe hepatomegaly, histological replacement and destruction of hepatic parenchyma by neoplastic lymphoid cells, diffuse CD79*α* immunoreactivity, absence of CD3 expression in neoplastic cells, and negative PCR results for the selected avian viral agents, the final diagnosis was hepatic B‐cell lymphoma in a common mynah (*A. tristis*).

All procedures were conducted in accordance with relevant animal welfare and veterinary diagnostic standards. The bird was euthanized humanely by a licensed veterinarian according to accepted avian veterinary practice and with the owner′s informed consent.

## 3. Discussion

This case describes hepatic B‐cell lymphoma in a common mynah (*A. tristis*). Hepatic lymphoma has been described far less frequently than epithelial hepatic tumors in birds, and most structured data on avian lymphoma are derived from psittacine reports [1, 3]. To the best of our knowledge, this is the first report of hepatic B‐cell lymphoma in a common mynah supported by immunohistochemistry.

Clinical signs in the present common mynah were nonspecific and mainly reflected severe hepatic enlargement and coelomic mass effect rather than a lymphoma‐specific presentation. The bird showed lethargy, altered vocalization, poor body condition, coelomic fluid accumulation, progressive ventral coelomic distension, and dyspnea, most likely because of massive hepatomegaly, displacement of adjacent viscera, and compression of the thoracoabdominal air sacs. An Indian ringneck parrot with multicentric T‐cell lymphosarcoma showed lethargy, poor body condition, hepatomegaly, visual impairment, and rapid clinical deterioration, whereas a yellow‐collared macaw with disseminated B‐cell lymphoma presented with reduced appetite, retro‐orbital involvement, and hepatic enlargement [4, 12]. In contrast, cutaneous B‐cell lymphoma in an umbrella cockatoo was characterized mainly by multifocal subcutaneous nodules and cutaneous plaques, emphasizing that the clinical phenotype of avian lymphoma is strongly influenced by tumor location [13]. The preserved appetite in the present case, despite poor body condition, should not be interpreted as evidence of mild disease, because advanced hepatic neoplasia may cause cachexia through tumor burden, hepatic dysfunction, fluid accumulation, and altered metabolism despite continued food intake. Weight loss with maintained or increased appetite has been reported in some birds with hepatic tumors, including cholangiocellular carcinoma [14]. Chronic ataxia and left‐leg lameness were also reported. Neurological signs have been described in avian lymphosarcoma cases without histological evidence of brain involvement, where hepatic encephalopathy or metabolic dysfunction was considered more likely than direct tumor infiltration [12].

Mynah birds pose a diagnostic challenge because hepatomegaly in these species is often interpreted in the context of iron storage disease or other metabolic hepatopathies. Previous reports of hepatic neoplasia in mynahs, including lymphosarcoma and hepatic lipomas in birds with concurrent iron storage disease, suggest that neoplastic and metabolic liver lesions may overlap clinically and grossly [2, 15]. In the present case, hemochromatosis was included in the imaging differential diagnosis; however, radiography and ultrasonography supported a hepatic space‐occupying process, considered most compatible with neoplasia, with severe hepatomegaly, coelomic mass effect, caudodorsal displacement of the intestinal loops, and compression of the thoracoabdominal air sacs. Similar diagnostic limitations have been reported in other avian coelomic and hepatic masses. In a common mynah with ovarian adenocarcinoma, ultrasonography helped distinguish a nonhepatic coelomic mass from apparent hepatomegaly [16], whereas in avian hepatic epithelial tumors, including cholangiocellular carcinoma, gross hepatic enlargement, and mass‐like lesions may suggest a malignant hepatic process, but tumor lineage requires histopathological confirmation [14, 16]. Therefore, imaging was essential for lesion localization and assessment of disease extent, but definitive classification as hepatic B‐cell lymphoma required histopathology and immunohistochemistry. A limitation of this case is the absence of special histochemical staining for hepatic iron, such as Prussian blue staining, which would have helped further assess concurrent iron storage disease.

The microscopic pattern in the present case differed from several previously reported avian hepatic and lymphoid lesions. In the greater Indian hill mynah reported by Hill et al., lymphosarcoma occurred concurrently with iron storage hepatopathy, and the liver showed portal fibrosis, bile duct proliferation, iron‐positive hepatocellular pigment, and pleomorphic mononuclear infiltrates extending from portal areas into sinusoids [15]. In contrast, the dominant lesion in the present common mynah was extensive and destructive replacement of hepatic parenchyma by densely packed lymphoid neoplastic cells, with effacement of hepatic cords and hepatocellular necrosis. This pattern supported lymphoma as the dominant process rather than iron storage hepatopathy alone. It also differed from primary epithelial hepatic tumors, such as cholangiocellular carcinoma, in which tubular, acinar, or solid epithelial arrangements would be expected rather than diffuse lymphoid infiltration [14]. The lesion shared similarities with the broader psittacine lymphoma series reported by Gibson et al., in which most round cell neoplasms showed infiltrative growth and hepatic involvement was common; however, that study also emphasized that cellular morphology alone was insufficient to reliably distinguish B‐cell from T‐cell lymphoma [3]. Extensive hepatic infiltration has also been described in disseminated B‐cell lymphoma in a yellow‐collared macaw, but that case involved multiple extrahepatic organs, whereas no histological evidence of tumor involvement was identified in the examined extrahepatic tissues in the present mynah [4]. In contrast to cutaneous B‐cell lymphoma in an umbrella cockatoo, where neoplastic lymphocytes formed dermal sheets and nodules without epithelial involvement, the present tumor was characterized by a destructive hepatic infiltrative pattern [13].

Immunophenotyping was necessary because avian round cell tumors may be difficult to classify on morphology alone, and the relationship between immunophenotype and biological behavior in avian lymphoma is still not well established [17]. In avian tissues, CD3 is generally considered the most reliable T‐cell marker, whereas B‐cell markers may show variable performance depending on species, antibody clone, fixation, and antigen‐retrieval conditions [3, 4, 8]. Although Pax5 and MUM‐1 have been emphasized as useful markers for B‐cell and plasma cell differentiation in psittacines, some avian round cell tumors may show limited or absent immunoreactivity despite appropriate controls, reflecting tumor biology, antigen loss, limited cross‐reactivity, or an undetermined immunophenotype. Therefore, interpretation should be based on staining distribution, cellular localization, morphology, and controls. In the present case, positive‐control bursal B cells showed strong CD20 and moderate CD79*α*/MB‐1 staining, confirming antibody reactivity; however, the hepatic neoplastic cells showed diffuse cytoplasmic CD79*α*/MB‐1 immunolabeling without diagnostic CD20 staining. This CD20‐negative/CD79*α*‐positive profile was interpreted cautiously and may reflect species‐related or clone‐dependent cross‐reactivity, antigen‐retrieval effects, tumor‐related CD20 antigen loss, or a differentiation state in which CD79*α*/MB‐1 is retained despite reduced CD20 expression [18, 19]. Accordingly, CD20 negativity was not considered sufficient to rule out B‐cell lineage. Together with the absence of CD3 expression in neoplastic cells, lack of mononuclear phagocyte markers, and negative TB and Giemsa metachromatic staining, the findings supported hepatic B‐cell lymphoma and excluded T‐cell, macrophage/histiocytic and mast cell origins [3, 13]. Additional markers such as Pax5 or MUM‐1 could further strengthen lineage confirmation in future cases; however, the present findings indicate that CD79*α* can be diagnostically informative in nonpsittacine birds when staining is diffuse, localized to the neoplastic population and concordant with the destructive hepatic lymphoid morphology.

Viral involvement was considered because lymphoid neoplasms in poultry are classically associated with oncogenic viruses, including Marek′s disease virus, ALV, and REV [17, 20]. In nonpoultry birds, however, the evidence remains inconsistent. A viral or retroviral association has been suggested in some avian lymphoma or tumor reports, including a European starling and budgerigars, whereas other cases, including a scarlet macaw, umbrella cockatoo, great horned owl, Humboldt penguin, pink‐backed pelican, Amazon parrots, little penguin, and silver gull, failed to establish a consistent viral association [13, 21–26]. In the present common mynah, PCR assays for Marek′s disease virus, ALV, and REV were negative. Therefore, the tested viral agents were not supported as causes in this case; however, these negative results do not definitively exclude viral involvement, including latent infection, viral load below the detection limit, untested oncogenic viruses, or nonviral mechanisms of lymphomagenesis.

Treatment of avian lymphoma remains poorly standardized, and reported protocols have produced variable outcomes [27]. In the present case, treatment was not attempted because of massive hepatic replacement, coelomic mass effect, respiratory compromise, and poor clinical condition. Similarly, in a yellow‐collared macaw with disseminated B‐cell lymphoma, only palliative therapy was used, including tramadol, meloxicam, and later prednisolone acetate; the bird died 2 months later [4]. An Indian ringneck parrot with multicentric T‐cell lymphosarcoma also deteriorated despite supportive and empirical antimicrobial treatment [12]. Taken together, these reports suggest that advanced visceral or multicentric lymphoma in birds may carry a poor prognosis, especially when severe hepatic involvement, coelomic mass effect, and respiratory compromise are present.

This case documents hepatic B‐cell lymphoma in a common mynah (*A. tristis*) and highlights that severe hepatomegaly in mynah birds should not be attributed solely to iron storage disease or metabolic hepatopathy. The absence of neoplastic infiltration in the examined extrahepatic tissues supported the liver as the only confirmed site of tumor involvement among the tissues examined, whereas negative PCR results did not support the involvement of the tested avian oncogenic viruses. Overall, this report adds to the limited knowledge of lymphoid neoplasia in common mynahs and emphasizes the use of combined clinical, imaging, pathological, and molecular evaluation in avian hepatic disease.

## Funding

No funding was received for this manuscript.

## Conflicts of Interest

The authors declare no conflicts of interest.

## Data Availability

The data that support the findings of this study are available from the corresponding author upon reasonable request.
